# Adam

**Published:** 1880-01

**Authors:** 


The Bistoury.

ELMIRA, N. Y., JAN., 1880.

The BISTOURY is published Quarterly, upon the first of April, July, October and January, at Fifty Cents a year in advance.

### ADAM.

The comments of the press upon the project of erecting a monument in this city, to our distinguished ancestor, the reputed father of us all—ADAM, has been widespread. Statues, monuments, and costly shafts have been erected to the memory of the "Father of our Country," and to our own immediate fathers, who have distinguished themselves in battle, in statesmanship, or by their inventive genius ; but the father of us all, but for whom none of these celebrated geniuses could have lived, nor the erection of monuments made possible for any of them, *he* remains unmonumented, without a stone or block of wood to signify that he ever existed.

It would be difficult to conceive of a person more entitled to monumental honors at the hands of his numerous and affectionate progeny, than this dear old father of ours, who, for centuries,—a lapse of time that no man can compute, has been shamefully neglected.

It has been left for Elmira to erect the first and only monument to Adam. Her enterprise, to say nothing of the genuine filial affection thus displayed, will, indeed already *has* gained for her a world wide exclamation of approval. A committee of distinguished men of this city—men of undoubted taste in matters of art, are in correspondence with the best sculptors of our country to procure a suitable design for a bronze figure which shall at once convey the idea of a primitive man. The statue will be a work of art that will not only be a delight to our own citizens, but shall possess such merit as to induce the visitations of gentlemen of culture and refinement from all parts of the world.

It has been asked by some newspapers, "why not erect a monument to Eve also ?

This is clearly done in the figure of Adam, for the word is an appellative noun, meaning the progenitors of the human race ; hence, Adam and Eve are both meant in the single term. In the only authentic history we have of Adam,—the Bible, we read :

"So God created man (Adam,) in his own image, in the image of God, created he, him ; male and female created he *them*." Then, it is recorded that Adam was created first, that he was caused to fall asleep (the first noted instance of the use of an anæsthetic in a surgical operation,) and that a rib was then taken from his side, and Eve made therefrom. This gives Adam priority over all comers, as the head and front of the human family.

It is not because of any special trait in Adam's character, or for any blessing entailed upon his race, that this monument is contemplated. As a gardner, he proved dilatory and inefficient, while he entailed untold misery upon his innocent descendents, by a disobediance of orders, engendered by a reckless stubborness. He was not even a brave man ; indeed, he exhibited a cowardice, in a certain apple transaction, perfectly inexcusable. For the same reason, he was not a gallant man, nor a model husband. From the very nature of his surroundings, and the early times in which he lived, he could not have been an orator, statesman, nor general. It is for none of these attributes therefore, that he is to be dignified with the compliment of a monument, but simply and solely because he is the grand progenitor of our race,—the great protoplast, to whom we owe our existence, and upon whose life and character we, his children, have so much improved as to make him more conspicuous than we are ourselves.

The monument will certainly be built. It will occupy a suitable place in our city. To its unveiling will come many of the scientific men of the world,—illustrious descendents of a primitive father. Profs. Huxley, Tindall, Darwin, and other men distinguished for their learning; Emperors, Kings, Queens and Princes of the old world, as well as exemplary citizens of our own country will be invited to the unveiling exercises. In short, it is intended that the entire affair shall be one in which all previous monumental celebrations for any person—anywhere, shall sink into utter insignificance. As soon as the design for the monument has been accepted and its site determined upon, we will apprise our readers of it, that they may be the better prepared to enjoy the work of art when they make their pilgrimage to the shrine of our long departed, neglected, yet revered and at last honored relative.

